# Impact of framing scheme optimization and smoking status on binding potential analysis in dynamic PET with [^11^C]ABP688

**DOI:** 10.1186/s13550-023-00957-8

**Published:** 2023-02-09

**Authors:** Cláudia Régio Brambilla, Jürgen Scheins, Lutz Tellmann, Ahlam Issa, Hans Herzog, N. Jon Shah, Irene Neuner, Christoph W. Lerche

**Affiliations:** 1 Institute of Neuroscience and Medicine, INM-4, Forschungszentrum Jülich GmbH, Jülich, Germany; 2 Department of Psychiatry, Psychotherapy and Psychosomatics, RWTH Aachen University, Aachen, Germany; 3 Institute of Neuroscience and Medicine, INM-11, Forschungszentrum Jülich GmbH, Jülich, Germany; 4 JARA – BRAIN – Translational Medicine, RWTH Aachen University, Aachen, Germany; 5 Department of Neurology, RWTH Aachen University, Aachen, Germany

**Keywords:** PET, Quantification bias, Non-displaceable binding potential, Framing scheme, mGluR5, Smoking status, Schizophrenia

## Abstract

**Background:**

For positron emission tomography (PET) ligands, such as [^11^C]ABP688, to be able to provide more evidence about the glutamatergic hypothesis in schizophrenia (SZ), quantification bias during dynamic PET studies and its propagation into the estimated values of non-displaceable binding potential (BP_ND_) must be addressed. This would enable more accurate quantification during bolus + infusion (BI) neuroreceptor studies and further our understanding of neurological diseases. Previous studies have shown BP_ND_-related biases can often occur due to overestimated cerebellum activity (reference region). This work investigates whether an alternative framing scheme can minimize quantification biases propagated into BP_ND_, whether confounders, such as smoking status, need to be controlled for during the study, and what the consequences for the data interpretation following analysis are. A group of healthy controls (HC) and a group of SZ patients (balanced and unbalanced number of smokers) were investigated with [^11^C]ABP688 and a BI protocol. Possible differences in BP_ND_ quantification as a function of smoking status were tested with constant 5 min (‘Const 5 min’) and constant true counts (‘Const Trues’) framing schemes. In order to find biomarkers for SZ, the differences in smoking effects were compared between groups. The normalized BP_ND_ and the balanced number of smokers and non-smokers for both framing schemes were evaluated.

**Results:**

When applying F-tests to the ‘Const 5 min’ framing scheme, effect sizes (η^2^p) and brain regions which showed significant effects fluctuated considerably with *F* = 50.106 ± 54.948 (9.389 to 112.607), *P*-values 0.005 to < 0.001 and η^2^*p* = 0.514 ± 0.282 (0.238 to 0.801). Conversely, when the ‘Const Trues’ framing scheme was applied, the results showed much smaller fluctuations with *F* = 78.038 ± 8.975 (86.450 to 68.590), *P* < 0.001 for all conditions and η^2^*p* = 0.730 ± 0.017 (0.742 to 0.710), and regions with significant effects were more robustly reproduced. Further, differences, which would indicate false positive identifications between HC and SZ groups in five brain regions when using the ‘Const 5 min’ framing scheme, were not observed with the ‘Const Trues’ framing.

**Conclusions:**

Based on an [^11^C]ABP688 PET study in SZ patients, the results show that non-consistent BP_ND_ outcomes can be propagated by the framing scheme and that potential bias can be minimized using ‘Const Trues’ framing.

## Background

Data from dynamic positron emission tomography (PET) acquisitions are often acquired in list-mode with high temporal granularity and are subsequently framed during the reconstruction process. This means that the acquired data are framed into certain time intervals and grouped in a sequence of images (dynamic image stack). In addition to the acquisition of the prompt events for each frame, it is also necessary to correct for several physical effects, such as randoms, scattered events, attenuation, and dead time. However, some of these corrections become more challenging at low count levels. This can become especially relevant during bolus + infusion (BI) studies with ^11^C radiotracers, where count rates become low toward the end of the acquisition leading to reconstructed frames with low counts. In the frame-based iterative reconstruction of the dynamic PET data, the reconstruction is performed independently for each frame. Once the dynamic frames are reconstructed, the activity of each voxel or selected volume of interest (VOI) can be obtained for each frame, resulting in a time activity curve (TAC) for each voxel or VOI.

The maximum likelihood-expectation maximization (MLEM) and the 3D ordinary poisson ordered-subset (OS) EM (3D OP-OSEM) reconstruction methods tend to be biased in regions with low activity concentrations and at low count rates. Furthermore, they tend to converge rather slowly for these regions. Thus, a very high number of iterations paired with long reconstruction times would be necessary to eliminate the positive bias [1]. Furthermore, VOIs can differ in convergence according to their shape, volume, and activity concentration; TACs with higher time resolution have shorter time intervals and, consequently, higher noise levels and quantification biases [1, 2]. This can be significant for BI studies as the time needed to reach equilibrium means that the count rate decreases during the study. Some of the reconstruction and image correction methods currently available may not sufficiently regularize noise and may introduce high variance during scatter correction at low counts [3, 4]. However, alternative reconstruction methods are emerging that aim to improve the quantification accuracy of the dynamic PET data [5–8]. Quantification bias for low counts during dynamic PET neuroreceptor studies and its propagation into the often-used outcome parameter non-displaceable biding potential (BP_ND_) is an important factor that must be addressed if accurate quantification is to be achieved. Previous studies have already shown BP_ND_-related biases of up to -15% due to overestimation of the cerebellum activity concentration (usually used as reference region for several neuroreceptor radiotracers and due to the fact that it shows low activity concentration and therefore low count rates during the acquisition) [2, 9–11].

As count rates get lower over the course of the BI study protocol, longer time frames are required to control image noise. Hence, one can use time frames of the same length throughout the protocol, or, since the frames after the tracer reaches equilibrium are the most important, non-constant intervals (increasing over the acquisition time) can be used until the tracer reaches equilibrium. At this point, the time frame is then fixed at a constant interval for the duration of the acquisition time (same time interval in the frames from equilibrium to the end of the acquisition time course). However, this choice is a trade-off between frame temporal resolution, counts in the frame (statistical noise), and quantification accuracy. Moreover, for multimodal studies with simultaneous PET/computed tomography (CT) or PET/magnetic resonance (MR), there is no consensus on how a framing scheme should be planned in order to facilitate enough temporal resolution to detect changes in BP_ND_. When the simple ratio method for analysis/quantification is the method of choice, reducing the bias caused by the low count rates, particularly in the reference region (e.g., cerebellum), is imperative to ensure a high level of accuracy.

The quantification bias described above may not only disturb the accurate detection of changes by stimulation or other challenges of the neuroreceptor system but may also impact biological confounders introduced by the investigated subjects.

To demonstrate these factors relating to quantification bias, patients with schizophrenia (SZ) and healthy controls (HC) underwent PET investigations with [^11^C]ABP688 [12], an allosteric antagonist for the metabotropic glutamatergic type 5 receptor (mGluR5), which may provide more evidence to elucidate the glutamatergic hypothesis in SZ.

An important confounder for [^11^C]ABP688 studies is the smoking status of the subject, as smoking has been shown to cause a global reduction in mGluR5 binding, as reported [13]. Furthermore, 60–90% of SZ patients are smokers [14], and it is known these individuals smoke more heavily and experience more difficulties in quitting smoking than smokers without SZ [15]. Further bias may arise from the fact that smokers might metabolize the radioligand faster in other organs (i.e., liver) than non-smokers, possibly influencing the uptake of [^11^C]ABP688 in the brain during BI protocol. As this might affect the count rate, the quantification bias and consequently the BP_ND_ results could also be impacted.

Other confounders may be gender and age. Although no significant effects of this kind were found in a study with healthy subjects [16], SZ patients presented different patterns between males and females [17]. Therefore, to eliminate this potential confounding factor, only male SZ patients (and HC) took part in this study.

The aim of this work was to evaluate the impact of the chosen framing scheme on BP_ND_ quantification and to show if an optimized frame scheme can minimize quantification bias. In addition, using the example of a SZ study with [^11^C]ABP688 as the radiotracer, we demonstrated the need to control confounders, such as smoking status, and the potential risk of misinterpreting the data due to the aforementioned influences on BP_ND_ quantification.

## Methods

### Subjects

Seventeen male subjects were studied for each group (SZ patients and HC) and were matched for age (SZ = 38.4 ± 10.6 and HC = 38.5 ± 11.4), level of smoking addiction, and education. All SZ patients were diagnosed with F20.0 paranoid SZ, except one patient who was diagnosed with F20.3 undifferentiated SZ. The patients in the SZ group had an illness duration of 19 ± 11 years. Subjects were instructed not to drink coffee or alcohol and not to take any medicine within 24 h before the measurement (with the exception of daily medication).

The study was approved by the Ethics Committee of the Medical Faculty at the RWTH Aachen University and the German Federal Office for Radiation Protection (Bundesamt für Strahlenschutz). Patients were recruited from Uniklinik RWTH Aachen and complied with the diagnostic criteria according to DSM-IV [18]. Informed consent was obtained for all subjects.

Three groups of SZ patients and HC were selected to represent different conditions for the analysis, as shown below:

#### Majority of smokers in the groups (unbalanced sample)

The effects of the smoking status on BP_ND_ quantification were evaluated in HC (a.1 and a.2) and SZ patients (b.1 and b.2) individually. Each group consists of nine smokers (S) and six non-smokers (nS), and therefore has a majority of smokers. To evaluate this effect, 2 nS were randomly excluded from each group of 17 SZ and 17 HC twice-represented by study cases called: A-2, A-3, B-2, and B-3. This unbalance mirrors the usual condition and the difficulty in finding nS SZ patients for such studies since smoking addiction is a known and relevant comorbidity of SZ. We tested the influence that the unbalance in the number of S in the analyzed groups (in two different cases for HC and SZ group selection) can have on BP_ND_ between and within the groups.

#### Equivalent number of smokers and non-smokers in the groups (balanced sample)

The effects of the smoking status on BP_ND_ quantification were individually evaluated in the HC and SZ groups again, but this time with the same number of S and nS in each group (8 S and 8 nS)-represented by case study A-1 and B-1) excluding one S per group (HC and SZ). As mentioned previously, the majority of published studies report a numerical balance of smoking status in SZ and HC, but usually with a majority of S in each group.

#### SZ versus HC-Equivalent number of smokers in the groups (balanced sample)

Balanced by smoking status, the samples were compared within and between group effects on BP_ND_. This analysis focused on potential differences in BP_ND_ caused by SZ.

#### HC versus SZ-Normalized BP_ND_ equivalent number of smokers in the groups (balanced sample)

Balanced by smoking status, the samples were compared, but this time normalized by differences in smoking status estimated from the temporal cortex regions. The focus of this analysis was on potential differences in the normalized BP_ND_ caused by SZ. The temporal cortex was chosen as the reference region for the smoking status as it shows a robust significant difference between S and nS, which was observed during this study in both groups for all kinds of evaluations of smoking effects on BP_ND_.

### PET/MR acquisition and PET BI protocol

Data were acquired with a hybrid 3 T MR-BrainPET insert [19] as reported in [12]. The PET data were acquired over 65 min in list-mode while applying a BI protocol (50% of the [^11^C]ABP688 activity was applied to the subject as a bolus and 50% was infused, not exceeding 600 MBq of injected activity per subject). PET and MR were synchronized during the acquisition so that the first 5–6 min of the MR resting data are acquired during a time where the equilibrium of [^11^C]ABP688 is reached (30 min after the bolus injection), thus enabling the evaluation of the BP_ND_ baseline for each subject. Smokers were not allowed to smoke in the two hours prior to the radiotracer injection.

The MR data acquisition started simultaneously with the PET bolus injection and the PET list-mode acquisition. Before the radiotracer reached equilibrium, a longitudinal relaxation time (T_1_) MPRAGE image was acquired for structural information with the following parameters: repetition time (TR) = 2250 ms; echo time (TE) = 3.03 ms; field of view (FOV) = 256 × 256 × 176 mm^3^, matrix size = 256 × 256 pixels, flip angle = 90°, 176 sagittal slices with 1 mm slice thickness, a generalized autocalibrating partially parallel acquisition (GRAPPA) factor of 2, and 70 auto-calibration signal lines. The structural MR image was also used for the PET attenuation correction based on the template method [20]. At about 30 min after the PET bolus injection, functional MR images (fMRI) were acquired using an effective transverse relaxation time (T_2_^*^) weighted echo planar image (EPI) sequences with the following parameters: TR = 2200 ms, TE = 30 ms, matrix size: 64 × 64 pixels with 36 slices and 3 × 3 x 3.75 mm^3^ voxel size. During the fMRI resting state (RS) scan, 180 functional volumes (approximately 5–6 min) were recorded for each subject. During this measurement, subjects were asked to lie down and keep their eyes closed without falling asleep. Only the time corresponding to the RS acquisition was chosen for the BP_ND_ analysis in this work.

### PET image reconstruction and BP_ND_ estimation

To compare the influence of the framing on the BP_ND_ bias, the data were reconstructed with two different framing schemes. In the first reconstruction, the list-mode data were sorted into 5 min frames (‘Const 5 min’). In the second reconstruction, the frames were reconstructed according to a method already presented [11, 21] with a constant number of true coincidences in each frame (‘Const Trues’), which minimizes and keeps the bias constant. For the ‘Const Trues’ scheme, PET true counts per frame based on this scheme were equivalent between subjects and also synchronized according to the fMRI RS acquisition moment. The subject with the lowest average of true counts registered a total of 9.35 × 10^6^ counts in the last 5 min of the acquisition. This value was taken as the fixed reference count per frame, and three interval time fractions from the subject where this low limit occurred were computed to give 0.267, 0.324, and 0.408 for the acquisition intervals. The intervals were computed for each subject according to the total length of the fMRI intervals and these fractions. The three frames within the time interval were then grown symmetrically around the center of these three intervals until the reference count (9.35 × 10^6^) was reached. Frames without matched counts and/or were not synchronized with fMRI were discarded for complete acquisition. In this way, all seventeen subjects had the same number of counts during the RS moments, and the reconstruction bias was equal for all time intervals.

For both reconstructions, corrections for attenuation [20], random and scattered coincidences, and dead time were applied. The 3D OP-OSEM [2] reconstruction was performed with 32 iterations and two subsets in a matrix of 256 × 256 pixels and 153 slices with a 1.25 mm^3^ isotropic voxel size. Post-processing was performed by filtering with a 2.5 mm 3D Gaussian filter. Head motion correction was performed with a multi-frame acquisition method (MAF) implemented in-house [22].

The frame(s) corresponding to the fMRI RS (starting around 30 min after the PET bolus injection and about 5 min in duration) were used for computing the BP_ND_ using the simple ratio method shown below (Eq. 1).

1 $$BP_{{{\text{ND}}}} = \left( {\frac{{C_{{\text{T}}} }}{{C_{{\text{R}}} }}} \right) - 1,$$

where *C*_T_ is the activity concentration in the target regions and *C*_R_ is the activity concentration in the reference region (cerebellum gray matter (GM)) after the radioligand reaches equilibrium and during fMRI RS.

The activity concentration was extracted using the Pmod v.3.9 PNEURO tool, and T_1_ MPRAGE images were used as the anatomical reference. The VOIs were applied in the subject’s PET space using the Hammer atlas [23]. The activity concentrations were extracted for 17 regions and the cerebellum GM, which was the reference region [17, 24–26]. The VOIs were: whole-brain GM, frontal left, frontal right, orbitofrontal cortex middle, parietal left, parietal right, temporal left, temporal right, temporal middle, primary auditory, anterior cingulate (ACC), posterior cingulate (PCC), caudate, putamen, thalamus, motor cortex and white matter.

### Statistical analysis

Statistical analysis was conducted with the Statistical Package v.25.0 (IBM SPSS Inc., Chicago, IL). The significance level was set to *P* = 0.05 for all tests, and all tests were two-tailed. Standard error (SE) bars were computed according to [27], which was implemented as a tool in the SPSS package. The SE bars were added by the SPSS toolbox to the BP_ND_ values calculated from PET image data. All analyzed brain regions were considered as a repeated variable (within-subjects) in the general linear model and, when appropriate, as between-subjects variables for the analysis of smoking status, SZ, and HC. Corrections for multiple comparisons were applied using the Bonferroni method, and the significance level was adjusted accordingly (*P* = 0.003). To evaluate the effect sizes of smoking status, the results from F-tests were also reported for each brain region in a pairwise comparison and when *P* < 0.05 for significant effects of smoking per region was reached.

For between-subject (HC and SZ) analyses, the regions from each group were compared using the general linear model, and the same significance level was assumed. Effect sizes were reported based on the partial eta squared (η^2^p), where 0.01 represents a small, 0.06 represents a medium, and 0.14 or higher represents a large effect size.

Despite having only 16 subjects per group (when groups were balanced with respect to the smoking status), the group size was considered sufficient based on available literature and standard power analysis. Previous comparable studies comprised the unique [^11^C]ABP688 PET study in SZ (*n* = 15 per group) at the time of the design of the study [17] and a ketamine study (one group of *n* = 10) [28]. Based on the sample size used in this study, between-subject effects were detected with a statistical power of around 80%, assuming repeated BP_ND_ measures for all analyzed brain regions for two groups with small to medium-size effects and *P* = 0.05. The estimations of the power were evaluated using GPower v.3.1 software.

## Results

### Balanced and unbalanced groups according to smoking status in BP_ND_ analysis

The effect sizes (η^2^p) were computed for smoking in each of the HC and SZ groups with both ‘Const 5 min’ and ‘Const Trues’ framing schemes, with standardized SE, and with an equivalent number of S and nS subjects. Table 1 shows a summary of the results for the different conditions of the groups arranged according to smoking status and framing schemes.

**Table 1:** Summary of all tested cases and correspondent results for smoking effects on BP_ND_

Framing scheme	Smoking status groups	*F*-test	*P*-value	η^2^p*
(a) Const 5 min	***A-1*** 16 HC (8 S and 8 nS)	9.389	0.005	0.238
	16 SZ (8 S and 8 nS)			
	***A-2*** 15 HC (9 S and 6 nS)	28.323	< 0.001	0.503
	15 SZ (9 S and 6 nS)			
	***A-3*** 15 HC (9 S and 6 nS)	112.607	< 0.001	0.801
	15 SZ (9 S and 6 nS)			
(b) Const Trues	***B-1*** 16 HC (8 S and 8 nS)	86.450	< 0.001	0.742
	16 SZ (8 S and 8 nS)			
	***B-2*** 15 HC (9 S and 6 nS)	79.076	< 0.001	0.739
	15 SZ (9 S and 6 nS)			
	***B-3*** 15 HC (9 S and 6 nS)	68.590	< 0.001	0.710
	15 SZ (9 S and 6 nS)			

^*^Effect size

Table 1 Analysis for both framing schemes with balanced (cases A-1 and B-1) and unbalanced groups (cases A-2, A-3, B-2 and B-3) with regard to smoking statuses in the subgroups. The results for the effect of smoking on BP_ND_ were obtained with the general linear model

Figure two shows all regions evaluated for BP_ND_ changes due to smoking status. The results are arranged according to the applied framing scheme and for the different test cases reported in Table 1. The regions that showed significant effects are marked by an asterisk on top of the green bars, which reports the effect size (η^2^p).

**Fig. 1:**
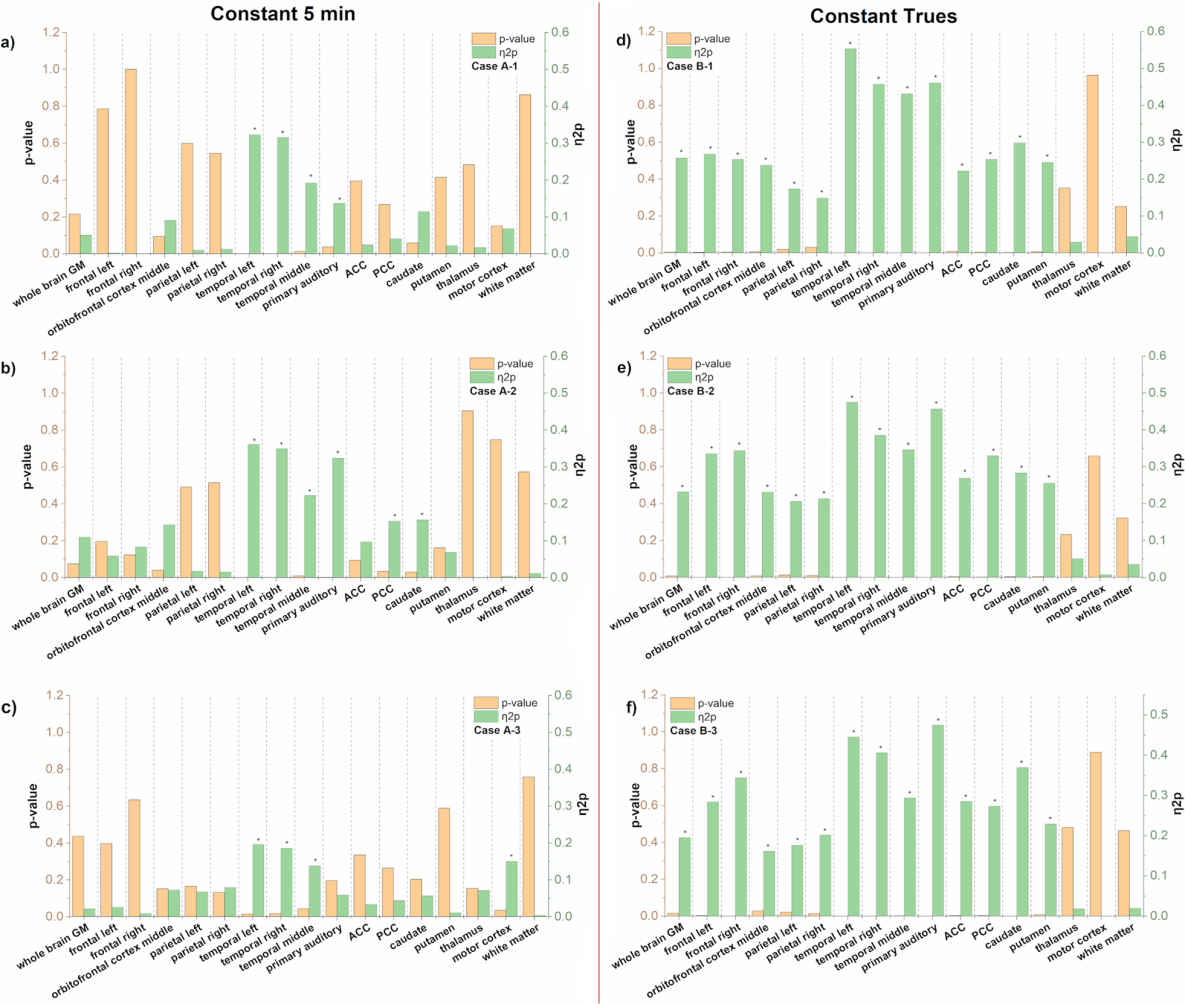
Regions depicted by significance and effect size according to test cases reported in Table 1. **a**–**c** Framed with ‘Const 5 min’ scheme **d**–**f** framed with ‘Const Trues’ scheme*.* **a** and** d** Cases A-1 and B-1 balanced samples by the number of S and nS, **b** and **e** Cases A-2 and B-2 unbalanced samples by the number of S and nS, **c** and **f** Cases A-3 and B-3 additional case of unbalanced samples by the number of S and nS. Here, 2 nS were randomly removed, and one additional S per group was added to simulate changes in the groups differing to cases A-2 and B-2 obtained with the same random method but differing in the nS removed from each group

The effect of smoking status per brain region was evaluated in a pairwise comparison. When unbalanced groups were analyzed, we observed an increased significance and larger effect size (as expected). Certainly, this did not dramatically change the conclusions about the smoking status affecting BP_ND_, but it is important to know how the significance and effects of a variable can influence the study in general and, in particular, how it influences the results in the involved brain regions. For case ***A-2***, a pairwise comparison between regions with both groups—HC and SZ, we observed a slightly changed scenario. In ***A-3***, we found an even larger effect size. This time, the regions that showed significant effects in the comparison were again different for other group compositions (Fig. 1 a and b). However, when the data were analyzed with the ‘Const Trues’ framing scheme (Fig. 1 d to f), we only observed a minor impact on smoking effect sizes between tested cases. Moreover, the F-tests were different to the corresponding results obtained with the ‘Const 5 min’ framing scheme when varying the composition of the groups. Furthermore, when using ‘Const Trues’ framing, the effect sizes and probabilities were reproduced more consistently in all three scenarios, B-1, B-2, and B-3.

### Balanced groups for the evaluation of the effect of smoking status on BP_ND_ per group (HC and SZ individually)

Another important question addressed in this study was whether smoking status affects the BP_ND_ in each group. As discussed in the Introduction, SZ patients find smoking cessation more difficult and usually smoke more heavily compared to non-SZ groups. It is, therefore, important to verify whether smoking status affects the BP_ND_ metrics in both groups unequally. Table 2 shows the regions affected by smoking status in HC (8 S and 8 nS) and SZ (8 S and 8 nS), and in the dependence of the framing scheme.

**Table 2:** Results for smoking status effects on BP_ND_ in different brain regions separated into HC and SZ groups and with framing schemes applied

Group	Framing scheme	Affected brain region	*F*-test	*P*-value	η^2^p*
HC	Const 5 min	Temporal right	5.867	0.030	0.295
		Motor cortex	4.677	0.048	0.250
SZ	Const 5 min	Temporal left	10.693	0.006	0.433
		Temporal right	7.551	0.016	0.350
		Temporal middle	5.039	0.041	0.265
		Caudate	4.849	0.045	0.257
HC	Const Trues	Temporal left	11.086	0.005	0.442
		Temporal right	9.471	0.008	0.404
		Temporal middle	7.010	0.019	0.334
SZ	Const Trues	Whole brain GM	7.019	0.019	0.334
		Frontal left	12.911	0.003	0.480
		Frontal right	18.371	< 0.001	0.568
		Parietal left	7.233	0.018	0.341
		Parietal right	13.766	0.002	0.496
		Temporal left	32.300	< 0.001	0.698
		Temporal right	20.491	< 0.001	0.594
		Temporal middle	16.253	0.001	0.537
		Primary auditory	35.933	< 0.001	0.720
		ACC	8.220	0.012	0.370
		PCC	18.291	< 0.001	0.566
		Caudate	10.589	0.006	0.431
		Putamen	9.192	0.009	0.396

*Effect size

Table 2 Groups balanced by smoking status, age, gender and education for the analysis of smoking status effects on BP_ND_ in HC and SZ grouped by framing scheme methods. The results from a pairwise analysis between subgroups (S and nS) for brain regions with significant differences reported

**Fig. 2:**
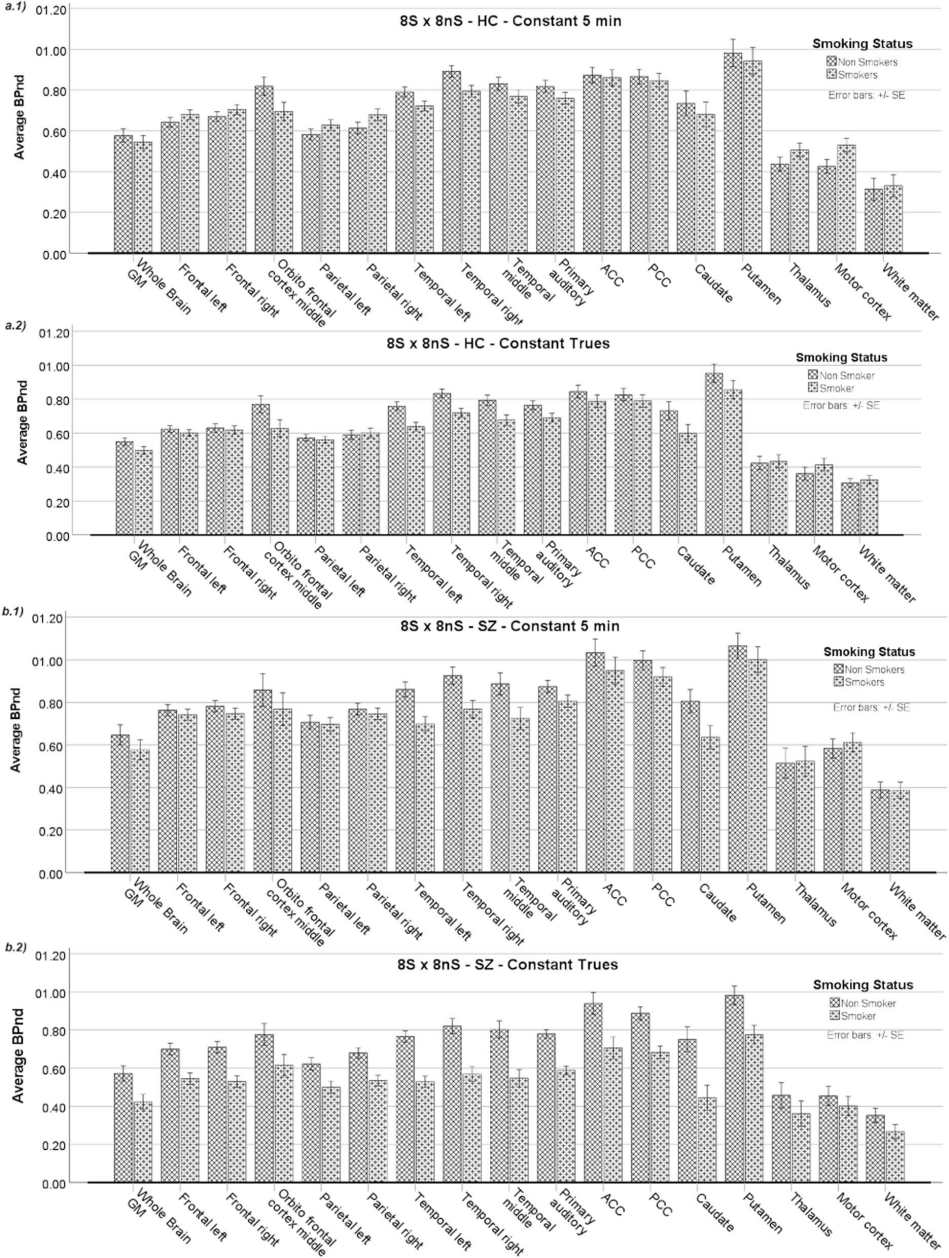
Smoking effects on BP_ND_ for HC and SZ groups showed individually for both framing schemes. The Average BP_ND_ for balanced subgroups according to smoking status is shown in: **a.1** For HC and ‘Const 5 min’ framing scheme, **a.2** For HC and ‘Const Trues’ framing scheme, **b.1** For SZ and ‘Const 5 min’ and **b.2** For SZ and ‘Const Trues’ framing scheme

It is evident that the SZ group showed more prominent effects, and more brain regions were affected by smoking status than the HC group. It must be expected that the outcome of the results is strongly influenced by the SZ group when smoking status was evaluated with both groups merged in the analysis. Interestingly, the temporal cortex showed significant differences for all methods and comparisons, indicating this mGluR5-rich region is heavily impacted by smoking habits. Figure 2 shows the regional average of the BP_ND_ for **a)** HC and **b)** SZ groups according to the smoking status and for the ‘Const 5 min’ and ‘Const Trues’ framing schemes. It should be noted that the ‘Const Trues’ scheme led to more brain regions with significant differences when comparing groups with different smoking statuses than in the case where the ‘Const 5 min’ scheme was applied.

### Balanced groups and BP_ND_ normalized to the temporal cortex: HC versus SZ comparison

In order to find biomarkers for SZ, the different effects of smoking status have to be considered for the comparison between subjects. As the temporal cortex always showed a significant effect of the smoking status on BP_ND_, independent of the framing scheme or balance of S and nS subjects in the groups, we normalized all other brain regions with respect to the average BP_ND_ of the three analyzed temporal cortex regions. In addition, we also compared the results for ‘Const 5 min’ and ‘Const Trues’ framing schemes. Table 3 provides a comparison of the groups for all investigated brain regions and both framing schemes.

**Table 3:** Results of the normalized BP_ND_ in the comparison between the HC and SZ groups separated by framing scheme

Brain region	Constant 5 min framing			Constant true counts framing		
	*F* test	*P*-value**	η^2^p*	*F* test	*P*-value**	η^2^p*
Whole Brain GM	1.401	0.247	0.048	0.326	0.572	0.012
Frontal left	12.036	0.002**	0.301	6.445	0.017	0.187
Frontal right	10.679	0.003**	0.276	2.469	0.127	0.081
Orbitofrontal cortex middle	0.710	0.407	0.025	2.556	0.121	0.084
Parietal left	10.712	0.003**	0.277	4.091	0.053	0.127
Parietal right	16.636	< 0.001**	0.373	5.205	0.030	0.157
Primary auditory	1.072	0.309	0.037	0.910	0.348	0.031
ACC	4.525	0.042	0.139	3.690	0.065	0.116
PCC	4.708	0.039	0.144	3.112	0.089	0.100
Caudate	0.044	0.835	0.002	0.253	0.619	0.009
Putamen	1.632	0.212	0.055	1.561	0.222	0.053
Thalamus	1.351	0.255	0.046	0.271	0.607	0.010
Motor cortex	10.829	0.003**	0.279	4.127	0.052	0.128
White matter	1.366	0.252	0.047	0.278	0.602	0.010

*Effect size, **Significance *P* ≤ 0.003 after Bonferroni correction are highlighted in bold italic

Table 3  Samples balanced by smoking status, age, gender and education for the analysis of normalized BP_ND_ based on different framing schemes. All brain regions with statistical analysis results were reported from a pairwise analysis between groups

For ‘Const 5 min’ framing, five regions showed significant differences, which was, however, not the case when applying the ‘Const Trues’ scheme. This shows how the uncontrolled bias of constant time framing can affect the results and subsequent interpretations. In this case, the false positive outcome would indicate an important significant difference between HC versus SZ in mGluR5 binding. No significant differences between the groups were observed when ‘Const Trues’ framing was used for bias minimization together with variance reduction in the data. In this case, the frontal left cortex was only significant before the Bonferroni correction. Thus, bias reduction and the control of confounders in the groups is highly relevant for the analysis of [^11^C]ABP688 PET in SZ studies.

## Discussion

In this work, we have demonstrated the influence of the framing scheme on reconstruction bias in a PET *in vivo* study with [^11^C]ABP688 for the first time. We have also shown how smoking status affects the BP_ND_ differently between regions by comparing SZ and HC groups. Considering that smoking addiction is a relevant comorbidity of SZ [29–35], the presented work confirms common limitations of studies on SZ that face the challenge of finding non-smokers for group comparisons with balanced groups demographics, and further shows the influence smoking status has on the data analysis in situations with a majority number of smokers in the study cohort. One of the limitations of this study was the subgroup number, with only eight smokers and non-smokers in each group (HC and SZ). It would be interesting to validate our findings by applying the same kind of analysis to a larger sample size of balanced and unbalanced numbers of smokers in the groups.

For the first time, an inter-subject comparison between framing schemes was performed that showed how framing scheme-dependent bias propagates into the BP_ND_.

Further, we demonstrated relevant changes in the results when our newly proposed ‘Const Trues’ framing scheme was used instead of the previous ‘Const 5 min’ framing scheme. As already shown in a previous publication [11], the ‘Const Trues’ framing scheme minimizes the reconstruction bias and keeps it constant during the BP_ND_ quantification. When applying the ‘Const 5 min’ framing scheme, the results varied more in terms of statistical significance, effect sizes, and brain regions affected by smoking status. This shows the potential of the ‘Const Trues’ framing scheme to minimize count fluctuations, to reduce the bias, and to obtain more consistent standard errors and variability.

Moreover, we also demonstrated the importance of evaluating the effects of smoking status on BP_ND_ in SZ and HC groups individually and showed that mGluR5 availability in SZ patients is more affected by tobacco than in HC when imaged with PET [^11^C]ABP688. Furthermore, potential false positive results, even when considering normalization for the different effects of smoking status over groups, were also presented, again showing the limitations caused by uncontrolled framing-dependent bias in the image reconstruction. When the comparison between SZ and HC groups was performed with normalized BP_ND_ (i.e., accounting for different smoking effects in both groups), false positive results were observed in the case of the ‘Const 5 min’ scheme but were not reproduced for the ‘Const Trues’ framing scheme. This means the strategy of normalizing the BP_ND_ by the region most affected by smoking status, i.e., the temporal lobe regions, was not useable as a biomarker for SZ, which significantly could differentiate the groups. However, it is possible that our basic assessment of smoking addiction (cigarettes consumption per day and years of consumption) unintentionally brought more variability into the analysis, and owing to the lack of a complete Fagerstrom scale from all participants and only a rudimentary dichotomous variable (S or nS) in the analysis, this may represent a limitation of the study. In light of this, it would be interesting to perform a deeper evaluation of tobacco addiction in an [^11^C]ABP688 study of SZ compared to HC in terms of the effects on BP_ND_. In addition to PET conventional quantification analysis, new methods are becoming available to aid the search for biomarkers in SZ. For example, radiomics is a method that has been tested in neuro-oncology [36] and is particularly interesting as it enables a great deal of additional information to be gathered during the analysis while simultaneously looking for the PET binding metrics and other feature-based metrics [37].

When ‘Const Trues’ framing was chosen, variations in the statistical significance, smoking effects, and effect size were minimized, and the regions affected by smoking status were reproduced when both groups were merged during the analysis. Once more, when evaluating HC and SZ groups individually, the effects of smoking status are more prevalent in a higher number of brain regions in SZ groups compared to HC groups.

We hypothesize that, due to the nature of the metabolization pathway, especially for subjects who smoke, the increased metabolization by other organs, such as the liver, leads to a reduced activity concentration of the radiotracer in the brain cortex (noted in the blood curves during the metabolite’s correction-data not shown). This might contribute to lower activity in the cortex and, in this case, a reduction in mGluR5 receptor binding. This would lead to a reduced count rate and reduced amount of events detected in the reconstructed image, thus causing increased variability in the image frame and bias in the BP_ND_ quantification. However, the results suggest this effect was minimized by the proposed ‘Const Trues’ framing scheme because the scheme keeps constant true events within the frames and, therefore, keeps the bias constant for the entire acquisition time course and inter- between-subjects frames. It is clear that other mechanisms could be responsible for this interplay of radiotracer metabolization, brain uptake and/or mGluR5 availability since the relationship between nicotine addiction and the functional role of mGluR5 down-regulation remains unclear, as already reported [13, 38]. Interestingly, smoking-related differences in BP_ND_ and the number of brain regions involved were strongly observed in SZ but were not observed in the corresponding HC group.

In light of these results, we strongly recommend the evaluation of the impact on smoking status in samples of SZ patients and HC groups prior to group analysis for other variables considered in the study. Similarly, in order to minimize biased results from the analysis in PET BI studies, we recommend an evaluation of the quantitative bias for the BP_ND_ prior to defining the framing scheme.

## Conclusions

This study shows how the framing scheme impacts reconstruction bias by demonstrating how bias propagation affects BP_ND_ metrics, consequently leading to inconsistent outcomes in a PET BI study on SZ with [^11^C]ABP688. In this preliminary investigation, potential false positive findings were minimized by applying the proposed ‘Const Trues’ framing scheme. Furthermore, the effect of additional confounding factors, such as smoking status in SZ, can be mitigated with this alternative framing scheme.

## Data Availability

The datasets used and/or analyzed during the current study are available from the corresponding author on reasonable request.

## Abbreviations

PET: Positron Emission Tomography
[^11^C]ABP688: (11)C-methyl iodide with the sodium salt of desmethyl-ABP688 (3-(6-methyl-pyridin-2-ylethynyl)-cyclohex-2-enone oxime)
SZ: Schizophrenia
BP_ND_: Non-Displaceable Binding Potential
BI: Bolus + Infusion
HC: Healthy Controls
Const 5 min: Constant 5 min
Const Trues: Constant True Counts
η^2^p: Effect Size
mGluR5: Metabotropic Glutamatergic Type 5 Receptor
VOI: Volume of interest
TAC: Time Activity Curve
MLEM: Maximum Likelihood-Expectation Maximization
3D OP-OSEM: Ordinary Poisson-Ordered Subset-Expectation Maximization
CT: Computed Tomography
MR: Magnetic Resonance
F20.0: Paranoid Schizophrenia Diagnose Classification
F20.3: Undifferentiated Schizophrenia Diagnose Classification
S: Smokers
nS: Non-Smokers
FOV: Field of View
MPRAGE: Magnetization Prepared-Rapid Gradient Echo
T_1_: Longitudinal Relaxation Time
TR: Repetition Time
TE: Echo Time
GRAPPA: Generalized Autocalibrating Partially Parallel Acquisition
EPI: Echo Planar Image
fMRI: Functional MR Images
T_2_^*^: Effective Transverse Relaxation Time
RS: Resting State
MAF: Multi-frame Acquisition Method
C_T_: Activity Concentration in the Target Regions
C_R_: Activity Concentration in the Reference Region
GM: Gray Matter
ACC: Anterior Cingulate Cortex
PCC: Posterior Cingulate Cortex
